# Supplementary material for: ArtSymbioCyc, a metabolic network database collection dedicated to arthropod symbioses: a case study, the tripartite cooperation in Sipha maydis
Source: mSystems. 2025 Mar 21;10(4):e00140-25. doi: 10.1128/msystems.00140-25 (PMC12013274; doi:10.1128/msystems.00140-25)
Supplement: Supplemental material — Figures S1 and S2; Tables S1 to S3. [file msystems.00140-25-s0001.pdf]

# Supplemental Material

**ArtSymbioCyc, a metabolic network database collection dedicated to arthropod symbioses: a case study, the tripartite cooperation in Sipha maydis** Patrice Baa-Puyoulet, Léo Gerlin, Nicolas Parisot, Sergio Peignier, François Renoz, Federica Calevro and Hubert Charles

**Table S1.** Databases currently contained in ArtSymbioCyc and specific features of the corresponding metabolic networks.

| Holobionts | Accession Numbers (genome reference) | Pathway numbers | Enzyme numbers | Reaction numbers | Compound numbers |
|---|---|---|---|---|---|
| Api-*Acyrthosiphon pisum* holobiont | - | 689 | 4,747 | 4,246 | 2,577 |
| Api-*Acyrthosiphon pisum* holobiont (*Buchnera* only) | - | 619 | 4,243 | 3,937 | 2,352 |
| Api-*Acyrthosiphon pisum* AL4f | GCF_005508785.2 (1) | 289 | 3,897 | 2,536 | 1,447 |
| Api-*Buchnera aphidicola* APS | GCF_000009605.1 (2) | 98 | 329 | 693 | 500 |
| Api-*Candidatus* Hamiltonella defensa T5A | GCF_000021705.1 (3) | 161 | 523 | 1,037 | 749 |
| Sma-*Sipha maydis* holobiont | - | 616 | 3,175 | 3,910 | 2,270 |
| Sma-*Sipha maydis* Midelt | GCA_034509805.1 (4) | 273 | 2,523 | 2,409 | 1,344 |
| Sma-*Buchnera aphidicola* Sm_Midelt | GCF_024029855.1 (5) | 58 | 221 | 473 | 321 |
| Sma-*Serratia symbiotica* Sm_Midelt | GCA_024160085.1 (5) | 135 | 410 | 899 | 617 |
| Cce-*Cinara cedri* holobiont | - | 619 | 3,903 | 3,994 | 2,387 |
| Cce-*Cinara cedri* | GCA_902439185.1 (6) | 274 | 3,426 | 2,458 | 1,488 |
| Cce-*Buchnera* aphidicola BCc | GCF_000090965.1(7, 8) | 41 | 189 | 402 | 320 |
| Cce-Serratia symbiotica Cc | GCA_000238975.1 (9) | 116 | 359 | 754 | 599 |
| Bta_MEAM1-*Bemisia tabaci* MEAM1 holobiont | - | 702 | 3,477 | 4,392 | 2,613 |
| Bta_MEAM1-*Bemisia tabaci* MEAM1 | MEAM1 V1.2 (10) | 295 | 2,442 | 2,584 | 1,491 |
| Bta_MEAM1-*Candidatus* Portiera aleyrodidarum | *Candidatus* Portiera[a] (10) | 33 | 131 | 286 | 213 |
| Bta_MEAM1-*Hamiltonella defensa* | *H. defensa*[a] (11) | 179 | 537 | 1060 | 712 |
| Bta_MEAM1-*Rickettsia sp.* | *Rickettsia sp.*[a] (12) | 117 | 363 | 784 | 534 |

| | | | | | |
|---|---|---|---|---|---|
| Bta_MED-*Bemisia tabaci* MED holobiont | - | 766 | 4,639 | 4,696 | 2,757 |
| Bta_MED-*Bemisia tabaci* MED | MED v1.0[b] (13) | 319 | 3,295 | 2949 | 1762 |
| Bta_MED-*Candidatus* Portiera aleyrodidarum BT-QVLC | GCA_000298385.1 (14) | 36 | 142 | 313 | 232 |
| Bta_MED-*Hamiltonella defensa* | GCF_000258345.2 (15) | 182 | 550 | 1206 | 768 |
| Bta_MED-*Candidatus* Cardinium hertigii | GCF_000689375.1 (16) | 74 | 241 | 669 | 386 |
| Bta_MED-*Wolbachia sp.* | GCF_900097055.1 (17) | 114 | 365 | 861 | 540 |
| Cle-*Cimex lectularius* holobiont | - | 564 | 4,322 | 3,863 | 2,241 |
| Cle-*Cimex lectularius* Harlan | GCF_000648675.2 (18) | 287 | 3,922 | 2,464 | 1,418 |
| Cle-*Wolbachia sp.* | GCF_000829315.1 (19) | 112 | 391 | 880 | 557 |
| Dme-*Drosophila melanogaster* holobiont | - | 783 | 6,402 | 4,466 | 2,715 |
| Dme-*Drosophila melanogaster* holobiont (gut microbiota) | - | 779 | 6,078 | 4,448 | 2,673 |
| Dme-*Drosophila melanogaster* | GCF_000001215.4 (20) | 296 | 3,857 | 2,333 | 1,307 |
| Dme-*Lactiplantibacillus plantarum plantarum NC8* | AGRI01000001.1 (21) | 232 | 846 | 1,379 | 1,001 |
| Dme-*Acetobacter pomorum* WJL DM001 | PRJNA60787 (22) | 286 | 1,241 | 1,612 | 1,179 |
| Dme-*Wolbachia sp.* | GCF_016584425.1 (23) | 108 | 340 | 735 | 549 |
| Gmo-*Glossina morsitans* holobiont | - | 676 | 3,429 | 3,943 | 2,360 |
| - Gmo-*Glossina morsitans Yale* | GmorY1[c] (24) | 284 | 2,258 | 2,273 | 1,370 |
| - Gmo-*Sodalis glossinidius morsitans* | GCF_000010085.1 (25) | 274 | 826 | 1,609 | 1,191 |
| - Gmo-*Wigglesworthia glossinidia (Yale colony)* | GCF_000247565.1 (26) | 134 | 349 | 824 | 608 |
| Phu-*Pediculus humanus corporis* holobiont | - | 575 | 2,329 | 3,760 | 2,132 |
| Phu-*Pediculus humanus* corporis USDA | GCA_000006295.1 (27) | 282 | 2,059 | 2,403 | 1,414 |
| Phu-*Candidatus* riesia pediculicola USDA | GCF_000093065.1 (27) | 105 | 287 | 786 | 494 |
| *Sitophilus oryzae* holobiont | - | 739 | 4,410 | 4,459 | 2,637 |
| *Sitophilus oryzae* Bouriz | GCF_002938485.1 (28) | 298 | 3,654 | 2,676 | 1,586 |
| *Candidatus* Sodalis pierantonius SOPE | GCF_000517405.1 (29) | 274 | 762 | 1,513 | 1,046 |

[a] http://www.whiteflygenomics.org/ftp/MEAM1/endosymbionts; [b] http://www.whiteflygenomics.org/ftp/MED/v1.0; [c] https://vectorbase.org/

**Table S2**. Organisms with GSMM available in the literature and used for our comparative analysis with ArtSymbioCyc reconstructions.

| **Organism** | **Short-name model (first author)** | **Reference** |
|---|---|---|
| *Acyrthosiphon pisum* (pea aphid) | Blow | (30) |
| *Buchnera aphidicola* of *A. pisum* | Blow | (30) |
| *Hamiltonella defensa* of *A. pisum* | Blow | (30) |
| *Bemisia tabaci* (whitefly) | Ankrah | (31) |
| *Portiera aleyrodidarum* of *B. tabaci* | Ankrah | (31) |
| *Hamiltonella defensa* of *A. pisum* | Ankrah | (31) |
| *Sodalis glossinidius* of *Glossina morsitans* | Belda | (32) |
| *Buchnera aphidicola* of *Cinara cedri* | Ponce-de-Leon | (33) |
| *Serratia symbiotica* of *Cinara cedri* | Ponce-de-Leon | (33) |
| *Drosophila melanogaster* (fruit fly) | Schönborn | (34) |
| *Drosophila melanogaster* (fruit fly) | Cesur | (35) |

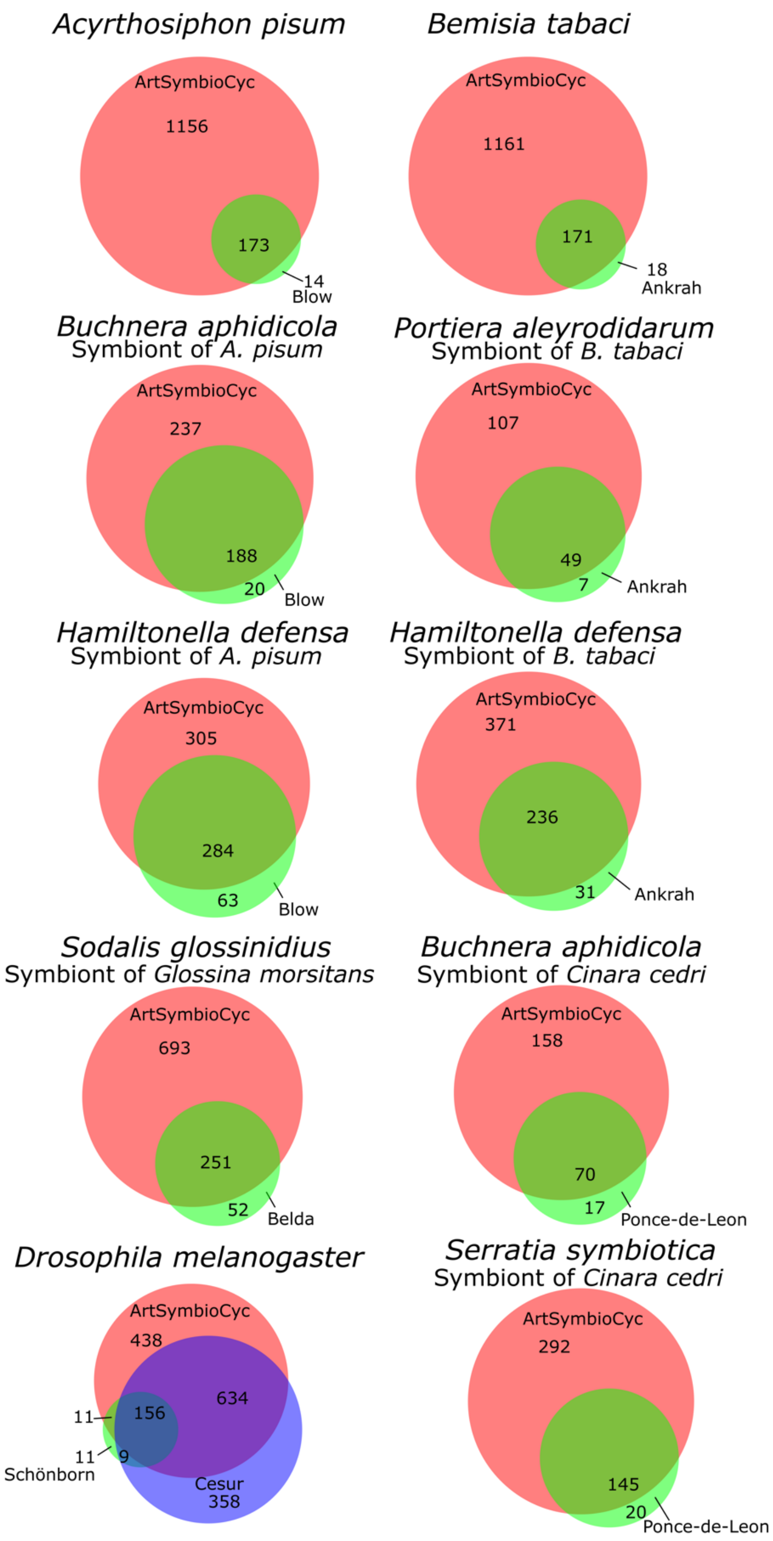


**Figure S1**. Venn diagrams for comparison of metabolic reconstructions of arthropods and their symbiotic bacteria. Red: metabolic reconstructions from the database ArtSymbioCyc, and in green or blue from GSMMs developed for FBA, with the first author of the associated article mentioned. See Table S2 for complete reference. EC numbers were used as a comparison metric, and the area is proportional to the number of EC numbers either specific or overlapping. Venn diagrams were made using the tool BioVenn https://www.biovenn.nl (36).

**Table S3**. Amino acids biosynthesis pathways comparison between the three aphid holobionts from the ArtSymbioCyc database collection: *Acyrthosiphon pisum* holobiont (Api-holobiont_bucap) with *Buchnera aphidicola* (Api-*B._aphidicola*_APS), *Cinara cedri* holobiont (Cce-holobiont) with *Buchnera aphidicola* (Cce-B_aphidicola_BCc) and *Serratia symbiotica* (Cce-S._symbiotica_Cc) and *Sipha maydis* holobiont (Sma-holobiont) with *Buchnera aphidicola* (Sma-B._aphidicola_Sm_Midelt) and *Serratia symbiotica* (Sma-S._symbiotica_Sm_Midelt). Green cells correspond to functional pathways automatically annotated and available to users from the interface. Red cells correspond to absences of automatically annotated pathways. Blue cells correspond to manually reconstructed pathways (the individual reactions are present but were not automatically gathered into pathways). Violet cells correspond to incomplete pathways manually tagged (pathways automatically annotated but with one or more lacking enzymes). The complete table can be directly visualized on the interface from this link.

| **Amino acid** | **Pathways** | *Api-B. aphidicola* APS | *Api-holobiont (bucap)* | *Cce-B. aphidicola* BCc | *Cce-S. symbiotica* Cc | *Cce-holobiont* | *Sma-B. aphidicola* Sm_Midelt | *Sma-S. symbiotica* Sm Midelt | *Sma-holobiont* |
|---|---|---|---|---|---|---|---|---|---|
| Gly | glycine biosynthesis I from serine | ✓ | ✓ | ✓ | ✓ | ✓ | ✓ | ✓ | ✓ |
| | glycine biosynthesis II (eukaryote glycine clivage complexe) | | ✓ | | | ✓ | | | ✓ |
| | glycine biosynthesis III from glyoxylate | | ✓ | | | ✓ | | | ✓ |
| | glycine biosynthesis IV from threonine | | ✓ | | | ✓ | | | ✓ |
| Ala | L-alanine biosynthesis II from pyruvate | | ✓ | | | ✓ | | | ✓ |
| | L-alanine biosynthesis III from Cysteine | ✓ | ✓ | ✓ | ✓ | ✓ | ✓ | ✓ | ✓ |
| Arg | L-arginine biosynthesis I (via L-ornithine) from glutamate | ✓ | n.f.[a] | from ornithine | | n.f. | from ornithine | | n.f. |
| Asn | L-asparagine biosynthesis I from aspartate | | ✓ | | | ✓ | | | ✓ |
| Asp | L-aspartate biosynthesis from oxaloacetate and glutamate | | ✓ | | | ✓ | | | ✓ |
| Cys | L-cysteine biosynthesis I from serine | ✓ | ✓ | | ✓ | ✓ | | | from HCYS[b] |
| Glu | L-glutamate biosynthesis I from glutamine | | ✓ | | | ✓ | | | ✓ |
| | L-glutamate biosynthesis II from 2-oxoglutarate | | ✓ | | | ✓ | | | ✓ |
| | L-glutamate biosynthesis IV from 2-oxoglutarate and gln | | ✓ | | | ✓ | | | ✓ |
| Gln | L-glutamine biosynthesis I | | ✓ | | | ✓ | | | ✓ |
| His | L-histidine biosynthesis from D-ribose-5-P | ✓ | ✓ | ✓ | | ✓ | ✓ | | ✓ |
| Ile | L-isoleucine biosynthesis I (from threonine) | | ✓ | | | ✓ | | | ✓ |
| Leu | L-leucine biosynthesis from 3-methyl-2-oxobutanoate | ✓ (incomplete)[c] | ✓ | ✓ (incomplete) | | ✓ | ✓ (incomplete) | | ✓ |
| Lys | L-lysine biosynthesis I from aspartate | ✓ | ✓ | ✓ | ✓ (incomplete) | ✓ | ✓ | ✓ (incomplete) | ✓ |
| Met | L-methionine biosynthesis from S-Methyl-L-Methionine | | ✓ | | | ✓ | | | ✓ |

| | | | | | | | | | |
|---|---|---|---|---|---|---|---|---|---|
| Phe | L-phenylalanine biosynthesis I from chorismate | ✓ (incomplete) | ✓ | ✓ (incomplete) | | ✓ | ✓ (incomplete) | | ✓ |
| | Chorismate biosynthesis from erythrose-4P | ✓ | ✓ | ✓ | | ✓ | ✓ | ✓ | ✓ |
| Pro | L-proline biosynthesis I (from L-glutamate) | | ✓ | | | ✓ | | | ✓ |
| Ser | L-serine biosynthesis I from 3P-D-glycerate | | ✓ | | | ✓ | | ✓ (incomplete) | ✓ |
| Thr | L-threonine biosynthesis from L-homoserine | ✓ | ✓ | ✓ | | ✓ | ✓ | | ✓ |
| | L-homoserine biosynthesis from aspartate | ✓ | ✓ | ✓ | | ✓ | ✓ | ✓ (incomplete) | ✓ |
| Trp | L-tryptophan biosynthesis from chorismate | ✓ | ✓ | | ✓ | ✓ | ✓ | | ✓ |
| | Chorismate biosynthesis from erythrose-4P | ✓ | ✓ | ✓ | | ✓ | ✓ | ✓ | ✓ |
| Tyr | L-tyrosine biosynthesis IV from phenylalanine | | ✓ | | | ✓ | | | ✓ |
| Val | L-valine biosynthesis from pyruvate | ✓ (incomplete) | ✓ | ✓ (incomplete) | | ✓ | ✓ (incomplete) | | ✓ |

[a] not found as an entire pathway per se but can be manually reconstructed (reactions are present); [b] homocysteine; [c] the pathway is incomplete (one or more enzymes are lacking).

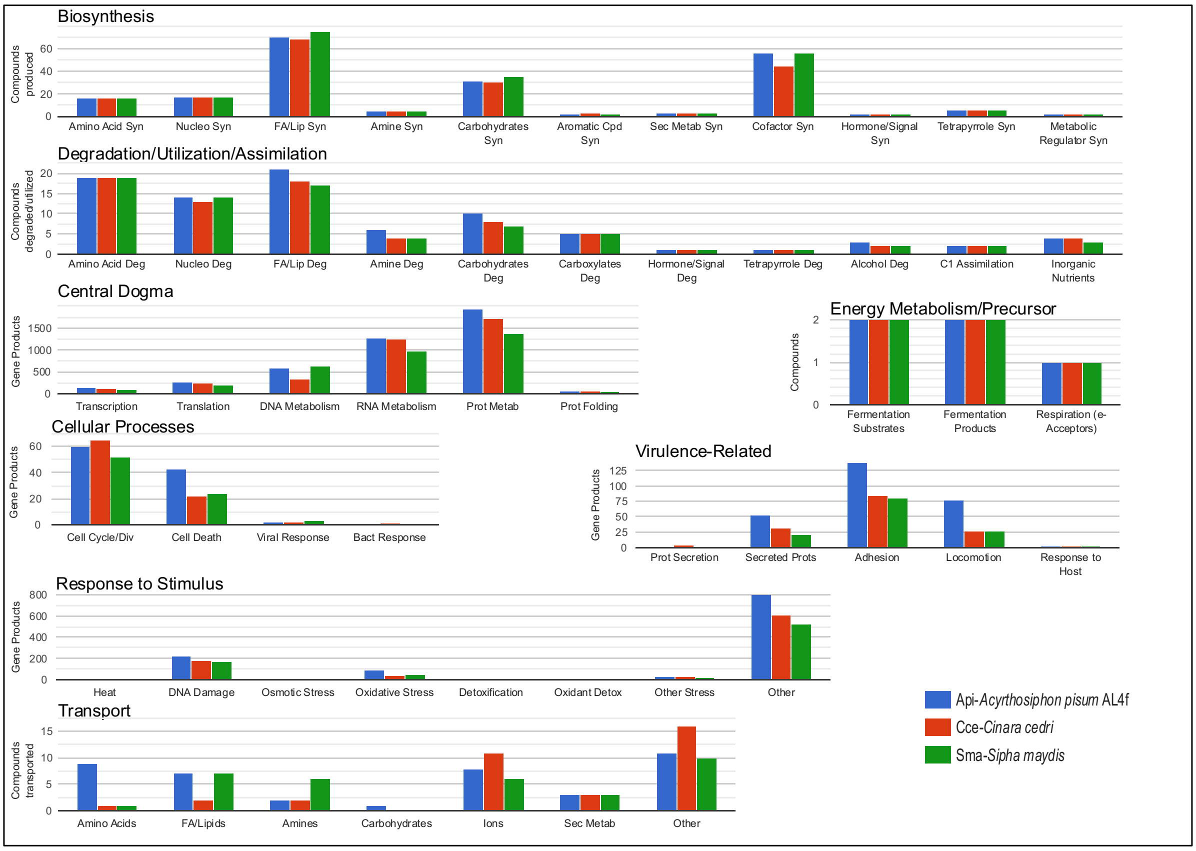


**Figure S2**. Central Metabolic pathway comparison between *A. pisum, C. cedri* and *S. maydis* using the Comparative Genome Dashboard (37). These comparisons can be visualized directly on the ArtSymbioCyc interface, and users can then click on each bar chart to see which pathways are specifically present or absent in the three organisms. As the plots are interactive, it is possible to access these pathways to determine the reactions and metabolites of which they are composed.

## References for the supplementary materials


1. Li Y, Park H, Smith TE, Moran NA. 2019. Gene family evolution in the pea aphid based on chromosome-level genome assembly. Mol Biol Evol 36:2143–2156.
2. Shigenobu S, Watanabe H, Hattori M, Sakaki Y, Ishikawa H. 2000. Genome sequence of the endocellular bacterial symbiont of aphids *Buchnera sp.* APS. Nature 407:81–86.
3. Degnan PH, Yu Y, Sisneros N, Wing RA, Moran NA. 2009. *Hamiltonella defensa*, genome evolution of protective bacterial endosymbiont from pathogenic ancestors. Proc Natl Acad Sci 106:9063–9068.
4. Renoz F, Parisot N, Baa-Puyoulet P, Gerlin L, Fakhour S, Charles H, Hance T, Calevro F. 2024. PacBio Hi-Fi genome assembly of *Sipha maydis*, a model for the study of multipartite mutualism in insects. Sci Data 11:450.
5. Renoz F, Ambroise J, Bearzatto B, Fakhour S, Parisot N, Lopes MR, Gala JL, Calevro F, Hance T. 2022. The Di-symbiotic systems in the aphids *Sipha maydis* and *Periphyllus lyropictus* provide a contrasting picture of recent co-obligate nutritional endosymbiosis in aphids. Microorganisms 10:1–21.
6. Julca I, Marcet-Houben M, Cruz F, Vargas-Chavez C, Johnston JS, Gómez-Garrido J, Frias L, Corvelo A, Loska D, Cámara F, Gut M, Alioto T, Latorre A, Gabaldón T. 2020. Phylogenomics identifies an ancestral burst of gene duplications predating the diversification of Aphidomorpha. Mol Biol Evol 37:730–756.
7. Perez-Brocal V, Gil R, Ramos S, Lamelas A, Postigo M, Michelena J, Silva F, Moya A, Latorre A. 2006. A small microbial genome: the end of a long symbiotic relationship? Science 314:312–313.
8. Gil R, Sabater-Muñoz B, Perez-Brocal V, Silva FJ, Latorre A. 2006. Plasmids in the aphid endosymbiont *Buchnera aphidicola* with the smallest genomes. A puzzling evolutionary story. Gene 370:17–25.
9. Lamelas A, Gosalbes MJ, Manzano-Marín A, Pereto J, Moya A, Latorre A. 2011. *Serratia symbiotica* from the Aphid *Cinara cedri*: a missing link from facultative to obligate insect endosymbiont. PLoS Genet.
10. Chen W, Hasegawa DK, Kaur N, Kliot A, Pinheiro PV, Luan J, Stensmyr MC, Zheng Y, Liu W, Sun H, Xu Y, Luo Y, Kruse A, Yang X, Kontsedalov S, Lebedev G, Fisher TW, Nelson DR, Hunter WB, Brown JK, Jander G, Cilia M, Douglas AE, Ghanim M, Simmons AM, Wintermantel WM, Ling K-S, Fei Z. 2016. The draft genome of whitefly *Bemisia tabaci* MEAM1, a global crop pest, provides novel insights into virus transmission, host adaptation, and insecticide resistance. BMC Biol 14:110.
11. Rao Q, Wang S, Su Y-L, Bing X-L, Liu S-S, Wang X-W. 2012. Draft genome sequence of "*Candidatus* Hamiltonella defensa," an endosymbiont of the whitefly *Bemisia tabaci*. J Bacteriol 194:3558–3558.
12. Rao Q, Wang S, Zhu D-T, Wang X-W, Liu S-S. 2012. Draft genome sequence of *Rickettsia sp.* strain MEAM1, isolated from the whitefly *Bemisia tabaci*. J Bacteriol 194:4741–4742.
13. Xie W, Chen C, Yang Z, Guo L, Yang X, Wang D, Chen M, Huang J, Wen Y, Zeng Y, Liu Y, Xia J, Tian L, Cui H, Wu Q, Wang S, Xu B, Li X, Tan X, Ghanim M, Qiu B, Pan H, Chu D, Delatte H, Maruthi MN, Ge F, Zhou X, Wang X, Wan F, Du Y, Luo C, Yan F, Preisser EL, Jiao X, Coates BS, Zhao J, Gao Q, Xia J, Yin Y, Liu Y, Brown JK, Zhou X "Joe", Zhang Y. 2017. Genome sequencing of the sweetpotato whitefly *Bemisia tabaci* MED/Q. GigaScience 6:1–7.
14. Santos-Garcia D, Farnier P-A, Beitia F, Zchori-Fein E, Vavre F, Mouton L, Moya A, Latorre A, Silva FJ. 2012. Complete genome sequence of "*Candidatus* Portiera aleyrodidarum" BT-QVLC, an obligate symbiont that supplies amino acids and carotenoids to *Bemisia tabaci*. J Bacteriol 194:6654–6655.
15. Rao Q, Rollat-Farnier P-A, Zhu D-T, Santos-Garcia D, Silva FJ, Moya A, Latorre A, Klein CC, Vavre F, Sagot M-F, Liu S-S, Mouton L, Wang X-W. 2015. Genome reduction and potential metabolic complementation of the dual endosymbionts in the whitefly *Bemisia tabaci*. BMC Genomics 16:226.
16. Santos-Garcia D, Rollat-Farnier P-A, Beitia F, Zchori-Fein E, Vavre F, Mouton L, Moya A, Latorre A, Silva FJ. 2014. The genome of *Cardinium* cBtQ1 provides insights into genome reduction, symbiont motility, and its settlement in *Bemisia tabaci*. Genome Biol Evol 6:1013–1030.
17. Selvaraj G, Santos-Garcia D, Mozes-Daube N, Medina S, Zchori-Fein E, Freilich S. 2021. An eco-systems biology approach for modeling tritrophic networks reveals the influence of dietary amino acids on symbiont dynamics of *Bemisia tabaci*. FEMS Microbiol Ecol 97:1–14.
18. Benoit JB, Adelman ZN, Reinhardt K, Dolan A, Poelchau M, Jennings EC, Szuter EM, Hagan RW, Gujar H, Shukla JN, Zhu F, Mohan M, Nelson DR, Rosendale AJ, Derst C, Resnik V, Wernig S, Menegazzi P, Wegener C, Peschel N, Hendershot JM, Blenau W, Predel R, Johnston PR, Ioannidis P, Waterhouse RM, Nauen R, Schorn C, Ott M-C, Maiwald F, Johnston JS, Gondhalekar AD, Scharf ME, Peterson BF, Raje KR, Hottel BA, Armisén D, Crumière AJJ, Refki PN, Santos ME, Sghaier E, Viala S, Khila A, Ahn S-J, Childers C, Lee C-Y, Lin H, Hughes DST, Duncan EJ, Murali SC, Qu J, Dugan S, Lee SL, Chao H, Dinh H, Han Y, Doddapaneni H, Worley KC, Muzny DM, Wheeler D, Panfilio KA, Vargas Jentzsch IM, Vargo EL, Booth W, Friedrich M, Weirauch MT, Anderson MAE, Jones JW, Mittapalli O, Zhao C, Zhou J-J, Evans JD, Attardo GM, Robertson HM, Zdobnov EM, Ribeiro JMC, Gibbs RA, Werren JH, Palli SR, Schal C, Richards S. 2016. Unique features of a global human ectoparasite identified through sequencing of the bed bug genome. Nat Commun 7:10165.

19. Nikoh N, Hosokawa T, Moriyama M, Oshima K, Hattori M, Fukatsu T. 2014. Evolutionary origin of insect – *Wolbachia* nutritional mutualism. Proc Natl Acad Sci 111:10257–10262.
20. Hoskins RA, Carlson JW, Wan KH, Park S, Mendez I, Galle SE, Booth BW, Pfeiffer BD, George RA, Svirskas R, Krzywinski M, Schein J, Accardo MC, Damia E, Messina G, Méndez-Lago M, De Pablos B, Demakova OV, Andreyeva EN, Boldyreva LV, Marra M, Carvalho AB, Dimitri P, Villasante A, Zhimulev IF, Rubin GM, Karpen GH, Celniker SE. 2015. The Release 6 reference sequence of the *Drosophila melanogaster* genome. Genome Res 25:445–458.
21. Axelsson L, Rud I, Naterstad K, Blom H, Renckens B, Boekhorst J, Kleerebezem M, Van Hijum S, Siezen RJ. 2012. Genome sequence of the naturally plasmid-free *Lactobacillus plantarum* strain NC8 (CCUG 61730). J Bacteriol 194:2391–2392.
22. Shin SC, Kim S-H, You H, Kim B, Kim AC, Lee K-A, Yoon J-H, Ryu J-H, Lee W-J. 2011. *Drosophila* microbiome modulates host developmental and metabolic homeostasis via insulin signaling. Science 334:670–674.
23. Duarte EH, Carvalho A, López-Madrigal S, Costa J, Teixeira L. 2021. Forward genetics in *Wolbachia*: Regulation of *Wolbachia* proliferation by the amplification and deletion of an addictive genomic island. PLOS Genet 17:e1009612.
24. International Glossina Genome Initiative, Attardo GM, Abila PP, Auma JE, Baumann AA, Benoit JB, Brelsfoard CL, Ribeiro JMC, Cotton JA, Pham DQD, Darby AC, Van Den Abbeele J, Denlinger DL, Field LM, Nyanjom SRG, Gaunt MW, Geiser DL, Gomulski LM, Haines LR, Hansen IA, Jones JW, Kibet CK, Kinyua JK, Larkin DM, Lehane MJ, Rio RVM, Macdonald SJ, Macharia RW, Malacrida AR, Marco HG, Marucha KK, Masiga DK, Meuti ME, Mireji PO, Obiero GFO, Koekemoer JJO, Okoro CK, Omedo IA, Osamor VC, Balyeidhusa ASP, Peyton JT, Price DP, Quail MA, Ramphul UN, Rawlings ND, Riehle MA, Robertson HM, Sanders MJ, Scott MJ, Dashti ZJS, Snyder AK, Srivastava TP, Stanley EJ, Swain MT, Hughes DST, Tarone AM, Taylor TD, Telleria EL, Thomas GH, Walshe DP, Wilson RK, Winzerling JJ, Acosta-Serrano A, Aksoy S, Arensburger P, Aslett M, Bateta R, Benkahla A, Berriman M, Bourtzis K, Caers J, Caljon G, Christoffels A, Falchetto M, Friedrich M, Fu S, Gäde G, Githinji G, Gregory R, Hall N, Harkins G, Hattori M, Hertz-Fowler C, Hide W, Hu W, Imanishi T, Inoue N, Jonas M, Kawahara Y, Koffi M, Kruger A, Lawson D, Lehane S, Lehväslaiho H, Luiz T, Makgamathe M, Malele I, Manangwa O, Manga L, Megy K, Michalkova V, Mpondo F, Mramba F, Msangi A, Mulder N, Murilla G, Mwangi S, Okedi L, Ommeh S, Ooi C-P, Ouma J, Panji S, Ravel S, Rose C, Sakate R, Schoofs L, Scolari F, Sharma V, Sim C, Siwo G, Solano P, Stephens D, Suzuki Y, Sze S-H, Touré Y, Toyoda A, Tsiamis G, Tu Z, Wamalwa M, Wamwiri F, Wang J, Warren W, Watanabe J, Weiss B, Willis J, Wincker P, Zhang Q, Zhou J-J. 2014. Genome sequence of the tsetse fly (*Glossina morsitans*): vector of african trypanosomiasis. Science 344:380–386.
25. Toh H, Weiss BL, Perkin SAH, Yamashita A, Oshima K, Hattori M, Aksoy S. 2006. Massive genome erosion and functional adaptations provide insights into the symbiotic lifestyle of *Sodalis glossinidius* in the tsetse host. Genome Res 16:149–156.
26. Rio RVM, Symula RE, Wang J, Lohs C, Wu Y, Snyder AK, Bjornson RD, Oshima K, Biehl BS, Perna NT, Hattori M, Aksoy S. 2012. Insight into the transmission biology and species-specific functional capabilities of tsetse (Diptera: Glossinidae) obligate symbiont *Wigglesworthia*. mBio 3:e00240-11.
27. Kirkness E, Haas BJ, Sun W, Braig HR, Perotti MA, Clark JM, Lee SH, Robertson H, Kennedy RC, Elhaik E, Gerlach D, Kriventseva E, Elsik C, Graur D, Hill CA, Veenstra JA, Walenz B, Tubío JM, Ribeiro JM, Rozas J, Johnston JS, Reese J, Popadic A, Tojo M, Raoult D, Reed DL, Tomoyasu Y, Kraus E, Krause E, Mittapalli O, Margam VM, Li HM, Meyer JM, Johnson RM, Romero-Severson J, Vanzee JP, Alvarez-Ponce D, Vieira F, Aguadé M, Guirao-Rico S, Anzola JM, Yoon KS, Strycharz JP, Unger MF, Christley S, Lobo NF, Seufferheld MJ, Wang N, Dasch GA, Struchiner CJ, Madey G, Hannick LI, Bidwell S, Joardar V, Caler E, Shao R, Barker SC, Cameron S, Bruggner RV, Regier A, Johnson J, Viswanathan L, Utterback TR, Sutton G, Lawson D, Waterhouse R, Venter JC, Strausberg R, Berenbaum MR, Collins FH, Zdobnov E, Pittendrigh BR. 2010. Genome sequences of the human body louse and its primary endosymbiont provide insights into the permanent parasitic lifestyle. Proc Natl Acad Sci U S A 107:12168–12173.
28. Parisot N, Vargas-Chávez C, Goubert C, Baa-Puyoulet P, Balmand S, Beranger L, Blanc C, Bonnamour A, Boulesteix M, Burlet N, Calevro F, Callaerts P, Chancy T, Charles H, Colella S, Da Silva Barbosa A, Dell'Aglio E, Di Genova A, Febvay G, Gabaldón T, Galvão Ferrarini M, Gerber A, Gillet B, Hubley R, Hughes S, Jacquin-Joly E, Maire J, Marcet-Houben M, Masson F, Meslin C, Montagné N, Moya A, Ribeiro De Vasconcelos AT, Richard G, Rosen J, Sagot M-F, Smit AFA, Storer JM, Vincent-Monegat C, Vallier A, Vigneron A, Zaidman-Rémy A, Zamoum W, Vieira C, Rebollo R, Latorre A, Heddi A. 2021. The transposable element-rich genome of the cereal pest *Sitophilus oryzae*. BMC Biol 19:241.

29. Oakeson KF, Gil R, Clayton AL, Dunn DM, Von Niederhausern AC, Hamil C, Aoyagi A, Duval B, Baca A, Silva FJ, Vallier A, Jackson DG, Latorre A, Weiss RB, Heddi A, Moya A, Dale C. 2014. Genome degeneration and adaptation in a nascent stage of symbiosis. Genome Biol Evol 6:76–93.
30. Blow F, Ankrah NYD, Clark N, Koo I, Allman EL, Liu Q, Anitha M, Patterson AD, Douglas AE. 2020. Impact of Facultative Bacteria on the Metabolic Function of an Obligate Insect-Bacterial Symbiosis. mBio 11:e00402-20.
31. Ankrah NYD, Luan J, Douglas AE. 2017. Cooperative Metabolism in a Three-Partner Insect-Bacterial Symbiosis Revealed by Metabolic Modeling. J Bacteriol 199.
32. Belda E, Silva FJ, Peretó J, Moya A. 2012. Metabolic networks of *Sodalis glossinidius*: a systems biology approach to reductive evolution. PLoS ONE 7:e30652.
33. Ponce-de-Leon M, Tamarit D, Calle-Espinosa J, Mori M, Latorre A, Francisco Montero, Pereto J. 2017. Determinism and contingency shape metabolic complementation in an endosymbiotic consortium. Front Microbiol 8:2290.
34. Schönborn JW, Jehrke L, Mettler-Altmann T, Beller M. 2019. FlySilico: Flux balance modeling of *Drosophila* larval growth and resource allocation. Sci Rep 9:17156.
35. Cesur MF, Basile A, Patil KR, Çakır T. 2023. A new metabolic model of *Drosophila melanogaster* and the integrative analysis of Parkinson's disease. Life Sci Alliance 6:e202201695.
36. Hulsen T, De Vlieg J, Alkema W. 2008. BioVenn – a web application for the comparison and visualization of biological lists using area-proportional Venn diagrams. BMC Genomics 9:488.
37. Paley S, Caspi R, O'Maille P, Karp PD. 2024. The comparative genome dashboard. Front Microbiol 15:1447632.
